# Preparation and Characterization of Softwood and Hardwood Nanofibril Hydrogels: Toward Wound Dressing Applications

**DOI:** 10.1021/acs.biomac.3c00596

**Published:** 2023-11-11

**Authors:** Yağmur Baş, Linn Berglund, Totte Niittylä, Elisa Zattarin, Daniel Aili, Zeljana Sotra, Ivana Rinklake, Johan Junker, Jonathan Rakar, Kristiina Oksman

**Affiliations:** † Division of Materials Science, Luleå University of Technology, SE-971 87 Luleå, Sweden; ‡ Umeå Plant Science Centre, Department of Forest Genetics and Plant Physiology, Swedish University of Agricultural Sciences, SE-901 87 Umeå, Sweden; § Laboratory of Molecular Materials, Division of Biophysics and Biotechnology, Department of Physics, Chemistry and Biology, Linköping University, SE-581 83 Linköping, Sweden; ∥ Center for Disaster Medicine and Traumatology, Department of Biomedical and Clinical Sciences, Linköping University, SE-581 85 Linköping, Sweden; ⊥ Department of Mechanical & Industrial Engineering (MIE), University of Toronto, Toronto, Ontario M5S 3G8, Canada

## Abstract

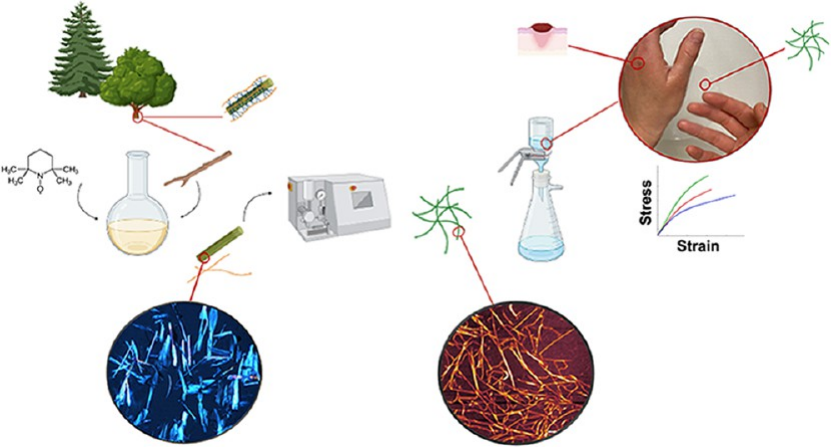

Hydrogels of cellulose nanofibrils (CNFs) are promising wound dressing candidates due to their biocompatibility, high water absorption, and transparency. Herein, two different commercially available wood species, softwood and hardwood, were subjected to TEMPO-mediated oxidation to proceed with delignification and oxidation in a one-pot process, and thereafter, nanofibrils were isolated using a high-pressure microfluidizer. Furthermore, transparent nanofibril hydrogel networks were prepared by vacuum filtration. Nanofibril properties and network performance correlated with oxidation were investigated and compared with commercially available TEMPO-oxidized pulp nanofibrils and their networks. Softwood nanofibril hydrogel networks exhibited the best mechanical properties, and *in vitro* toxicological risk assessment showed no detrimental effect for any of the studied hydrogels on human fibroblast or keratinocyte cells. This study demonstrates a straightforward processing route for direct oxidation of different wood species to obtain nanofibril hydrogels for potential use as wound dressings, with softwood having the most potential.

## Introduction

Cellulose nanofibrils (CNFs) are fibrils with diameters 4–20 nm and lengths 0.5–2 μm^1^ and with optical transparency^2^ and high mechanical strength.^3^ They offer applications in drug delivery systems,^4,5^ tissue substitutes and scaffolds,^6^ and wound dressings.^7,8^ CNFs are isolated from various sources such as plants, bacteria, and wood, and among these, wood is the most abundant and exploited CNF source.^9^ In woody mass, fibril bundles comprised of CNFs are embedded in a matrix of hemicelluloses and lignin in the cell wall,^10^ and the isolation of CNFs from wood requires energy input and processing, where chemical or mechanical treatments are applied for liberation of nanofibrils.^11^ A common approach to produce CNFs is through 2,2,6,6-tetramethylpiperidine-1-oxyl (TEMPO)-mediated oxidation followed by defibrillation of cellulose, resulting in anionically charged CNFs with widths smaller than 20 nm and lengths around a couple of micrometers.^12^ Introduction of negative charges on the fiber surface has found use in many applications as it eases the fibrillation by electrostatic repulsion while influencing the CNF network properties by enhancing interfibrillar interactions. Conventionally, TEMPO-oxidation is applied on cellulose isolated from wood through pulping and bleaching steps,^12^ where its native properties like the degree of polymerization (DP) and crystallinity are afflicted because of such processes.^13,14^ New mechanisms were devised to preserve DP in TEMPO-oxidation of cellulose^15^ although the pulping step was never obviated. To this end, we have studied the possibility of preserving the native characteristics of CNFs in woody mass *via* direct mild TEMPO-mediated oxidation of a hardwood specie, namely aspen.^16^ Herein, CNFs were obtained *via* simultaneous delignification and oxidation of wood in a one-pot pretreatment, followed by fibrillation. We also studied the effect of direct mild TEMPO-oxidation of tension and normal wood of aspen, where average toughness (11.6 and 3 MJ m^–3^, respectively) and strain at break (11% and 3%, respectively) differed significantly for their corresponding CNF networks in relation to their source.^17^ However, these studies were performed using a hybrid aspen wood supplied by SweTree Technologies AB (Umeå, Sweden), which was field grown and is not currently commercially available. Therefore, mild TEMPO-oxidation of commercially available raw materials that are hardwood (HW) and softwood (SW) particles and their comparison to commercial TEMPO-oxidized pulp are of interest for large-scale production.

The properties of CNFs are related to the characteristics of the wood cell wall, associated with the native cellulose, hemicellulose, and lignin compositions and the processing of the CNF source. The hemicellulose and lignin composition and thereby the structure differ remarkably between SW and HW. Galactoglucomannan is the primary hemicellulose in SW, whereas glucuronoxylan is the principal hemicellulose in HW.^18^ The main lignins in SW and HW are guaiacyl and guaiacyl syringyl lignin, respectively, with the former having a higher molecular weight than the latter. In addition, the anatomy of SW is distinct from that of HW due to their different cell types and native fiber lengths.^19^ TEMPO-oxidation of different wood celluloses obtained after pulping and bleaching has been studied widely.^12,14,15,20−23^ To our knowledge, only a few studies have produced CNFs directly from wood using TEMPO-mediated oxidation^16,17,24,25^ and investigated the resulting fiber properties, where all focused only on HW species. No publication comparing the properties of nanofibrils derived from directly mild TEMPO-oxidized commercially available SW and HW, and their network formation into hydrogels, has come to our attention.

Hydrogels are three-dimensional (3D) polymeric networks with high water absorption capability and have been widely used as wound dressing materials, as they provide a moist environment crucial for wound healing. Ideally, dressings should possess mechanical stability to protect the wound from damage, act as a barrier against pathogens, perform the absorption of exudates, and should be nontoxic, hypoallergenic, easily removable, and cost-efficient.^26^ Hydrogels of CNF networks are promising wound dressing materials owing to their biocompatibility, high water absorption, and mechanical properties.^27^ The cytotoxicity and immunogenicity of CNFs have been studied through applications with various cell types,^28,29^ and examples of dressings of bacterial cellulose (BC),^30^ that is the biosynthetic source of cellulose,^31^ are available in the market, *e.g.*, Epiprotect.^32,33^ Reports on mechanically treated wood CNF hydrogels for wound dressings are present in the literature,^34^ and such products are also being marketed, *e.g.*, FibDex.^35^ In addition, evaluation of TEMPO-oxidized CNF gels for cytotoxicity and skin applicability for their potential use in wound care-related applications gave promising results.^36,37^ Previously, we have shown that hydrogels of CNFs obtained from direct mild TEMPO-oxidation of field-grown aspen can mimic the properties of BC-derived hydrogels.^38^ It is now our interest to develop and produce CNF dressings by directly utilizing two different wood species starting from commercial raw materials and compare their properties toward potential applications, in order to provide wound dressings by feasible processing of various sources of woody biomass.

In this study, CNFs were obtained directly from SW and HW, while avoiding pulping and bleaching steps, by means of TEMPO-oxidation under mild conditions (pH = 6.8). Oxidized SW and HW were fibrillated using a microfluidizer, and the chemical composition, carboxylate contents, and nanofibril properties were investigated to understand the role of the wood source in relation to CNFs and their networks as hydrogels. Nanofibrils were assembled into networks *via* vacuum-assisted filtration and water absorption capacities; mechanical and thermal properties were studied. Oxidized SW and HW nanofibrils and networks were compared to those obtained from commercially available TEMPO-oxidized cellulose (TO-C) that was processed and assembled using the same conditions. Furthermore, oxidized SW nanofibrils (TO-SWNFs) and oxidized HW nanofibrils (TO-HWNFs) were evaluated with toxicological risk assessment and found to be suitable for potential applications as wound dressings. Production of CNFs directly from wood with well-performing material properties applicable to different commercially available wood species is interesting from industrial and sustainability perspectives.

## Experimental Section

### Materials

Softwood (BK 40–90, average size 1–2 mm, mainly spruce) and hardwood (HBS 150–500, average size 300–500 μm, mainly beech) particles, namely Lignocel, were purchased from J. Rettenmaier & Söhne GMBH (Rosenberg, Germany) and were stored at room temperature (RT). Commercial TEMPO-oxidized softwood Kraft pulp was purchased from Nippon Paper Ind. Co. (Tokyo, Japan) as never-dried pulp and stored at +4 °C in a refrigerator prior to use. TEMPO catalyst, sodium hypochlorite (NaClO), sodium chlorite (NaClO_2_), sodium phosphate dibasic (Na_2_HPO_4_), and sodium hydroxide (NaOH) were bought from Sigma-Aldrich (Darmstadt, Germany). Sodium phosphate monobasic (NaH_2_PO_4_) was bought from G-Biosciences (St. Louis, MO).

### TEMPO-Oxidation of Wood

SW and HW were oxidized using a TEMPO/NaClO/NaClO_2_ system in phosphate buffer at pH = 6.8 using 55.1 mmol of NaClO_2_, 4 mmol of NaClO, and 0.10 mmol of TEMPO per gram of dry wood as described in a previous study,^16^ with slight modification. The wood/liquor ratio was kept as 1 g of wood in 100 mL^–1^ phosphate buffer. Wood particles (20 g, dry weight) were soaked in the reaction buffer for 24 h prior to oxidation. TEMPO (320 mg, 2.05 mmol) and NaClO_2_ (100 g, 1105 mmol) were dissolved in wood suspension in a 5 L flask in the presence of the buffer. The flask was stirred in an oil bath at 60 °C for 1 h. NaClO (2M, 6–14%, 40 mL) was added to reaction media, and the flask was stoppered and stirred for a total of 72 h at 60 °C. The nonsoluble fractions after oxidation were washed with dH_2_O until a neutral pH was reached for water used for washing. TEMPO-oxidized SW and HW are named TO-SW and TO-HW, respectively. The reference material used in this study is TEMPO-oxidized cellulose and is abbreviated as TO-C.

### Nanofiber Preparation

TO-SW, TO-HW, and TO-C fiber slurries were fibrillated using a high shear processor LM20 Microfluidizer (Microfluidics International Corp., Westwood, MA) equipped with H10Z and H30Z interaction chambers (minimum internal dimensions 100 and 200 μm, respectively) and monitored for one pass at 1000 bar pressure. Following the washing, consistencies of TO-SW and TO-HW fibers were diluted to 0.75 wt %. The TO-C fiber slurry was prepared based on the dry fiber content of pulp, and its pH was controlled at 8.5–9 by addition of 1 M NaOH solution to obtain COO^–^Na^+^ type fibers. All slurries were prepared in dH_2_O and stirred at RT for 24 h at 500 rpm (IKA RCT basic, Ø 135 mm) prior to fibrillation. Nanofibrils obtained upon the processing of TO-SW, TO-HW, and TO-C are named TO-SWNF, TO-HWNF, and TO-CNF, respectively, and were stored at 4 °C prior to use.

### Preparation of Networks

TO-SWNF, TO-HWNF, and TO-CNF networks were prepared according to a precalculated grammage of 20 g m^–2^, based on dry fiber weight. For the preparation of 90 mm diameter circular networks, nanofibril suspensions with 0.25 wt % consistency were prepared using a magnetic stirrer for 30 min at 500 rpm (IKA RCT basic, Ø 135 mm) at RT. The suspension was poured on a Durapore hydrophilic PVDF membrane 0.1 mm (Merck Life Sciences AB, Solna, Sweden) located in a sintered glass funnel connected to a vacuum pump VCP80 (VWR International AB, Stockholm, Sweden). The fibril suspension was filtered until the majority of dH_2_O was removed, and a wet cake was obtained. The wet cake was placed in metal meshes surrounded by paper tissue located in between two metal plates to dry in ambient conditions under weights (5.3 kg, circular) for 24 ± 1 h. Dry networks were placed between Mylar films and pressed using a hot-press LabEcon 300 (Fontijne Grotnes, Vlaardingen, The Netherlands) at 225 kPa and 100 °C for 30 min before tensile testing.

### Conductometric Titration

Carboxylate content of the oxidized wood nanofibrils and commercial cellulose nanofibrils was determined according to a previously described conductometric titration method with slight modification.^22,25^ 0.1 M hydrochloric acid and 0.01 M sodium chloride were added to 125 mL of respective nanofibril suspensions (≈0.24 wt %) for protonation until the CNF suspension pH reached around 2–3. Conductometric titration was carried out using an Eco titrator (Metohm Nordic AB, Bromma, Sweden) starting from pH 2 to 3 until the pH reached 11, using 0.04 M NaOH solution with an addition rate of 0.1 mL min^–1^. Carboxylate groups were quantified from the titration curves according to eq 1. Tangent lines were drawn in Tiamo software (Metohm Nordic AB, Bromma, Sweden) to calculate the volume of NaOH consumed. Three titrations were performed for each sample, and the calculated average with standard deviation is reported as mmol g^–1^ of the dry material.
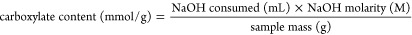
1

### Yield

The mass yield of TEMPO-oxidation of wood species was calculated according to eq 2, where Wi is the initial dry weight of wood and Wf is the final dry weight of TEMPO-oxidized fibers. Yields were reported as a calculated average of three measurements with standard deviations.
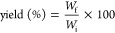
2

### Chemical Composition Analysis

SW and HW were treated according to a procedure modified from Gandla et al.^39^ to remove soluble sugars, protein, phenolics, pigments, and starch prior to all chemical composition assays to avoid artifacts. TO-SW, TO-HW, and TO-C were not subjected to any pretreatment. The crystalline cellulose amount (wt %) in the cell wall of SW and HW, and of TO-SW and TO-HW, was determined by Updegraff cellulose assay.^40^ Extracted crystalline cellulose was hydrolyzed in 72% sulfuric acid,^41^ and the glucose unit was quantified using anthrone colorimetric assay^42^ from the absorbance measured at 620 nm using a microplate spectrophotometer (BioTek, Winooski, VT).

Lignin in SW and TO-SW was quantitatively analyzed using the protocol based on Foster et al.^43^ Briefly, lignin was solubilized with acetyl bromide and acetic acid, and solubilized lignin unit aromatic rings were quantified from absorbance values at 280 nm using an ultraviolet–visible (UV–vis) spectrophotometer. The % (w/w) lignin was calculated using a commercially available extracted kraft lignin (Sigma-Aldrich 370959) as standard. Lignin in HW and TO-HW was analyzed using the Klason lignin method,^44^ and total lignin was reported as the sum of Klason and acid-soluble lignins. Lignin in TO-C was qualitatively analyzed using pyrolysis gas chromatography–mass spectrometry (GC–MS) as % (w/w) (EGA/PY-3030D and AS-1020E, Frontier Lab, Japan; 7890*A*/5975C, Agilent Technologies, Santa Clara, CA) as percentage of the total peak area.^45^

Monosaccharide composition analysis of dry fine wood powders (SW and HW, 500 μg ± 10%) that was pretreated as above, or untreated TO-SW and TO-HW and 30 μg of inositol used as internal standard, together with standards of nine monosaccharides (Ara, Rha, Fuc, Xyl, Man, Gal, Glc, GalA, and GlcA, each at 5, 10, 20, 50, and 100 μg) were methanolized and derivatized, and its silylated monosaccharides were separated in GC/MS (7890*A*/5975C; Agilent Technologies, Santa Clara, CA).^39,46,47^ Raw data MS files from GC/MS analysis were converted to NetCDF format in Agilent Chemstation Data Analysis (Version E.02.00.493) and exported to the RDA (version 2016.09; Swedish Metabolomics Centre (SMC), Umeå, Sweden). Data pretreatment procedures, such as baseline correction and chromatogram alignment, peak deconvolution, and peak integration followed by peak identification were performed in RDA. 4-O-methylglucuronic acid was identified according to a previously described method.^48^

### Polarized Optical Microscopy

Polarized optical microscopic images of TO-SW, TO-HW, and TO-C fibers before and after fibrillation were taken using a Nikon Ci Eclipse LV100N POL (Nikon, Kanagawa, Japan) using NIS-Elements D 4.30 imaging software, at RT with consistencies of 0.5 wt %.

### Viscosity Measurement

Viscosity values were measured using a Vibro-Viscometer SV-10 (A&D Company, Tokyo, Japan) at 0.75 ± 0.05 wt % concentrations at 21 °C. Samples were measured as triplicate for a duration of 3 min each after the plateau was reached, and the average viscosity values were reported with standard deviations.

### Scanning Electron Microscopy

Fiber dispersions of TO-SW, TO-HW, and TO-C after stirring at 0.25 wt % consistencies were drop cast on holders and dried at RT. Samples were sputtered with 15 nm of a platinum layer using an EM ACE200 (Leica, Wetzlar, Germany) to reduce electron charging. SEM images were recorded using a JEOL JSM-6460LV (Jeol Ltd., Tokyo, Japan) operating at an acceleration voltage of 10 kV. For the network surface images, 4 mm × 4 mm rectangular networks were cut from the middle sections of circular networks and observed on holders. Fiber size and length were measured using ImageJ (National Institutes of Health and the Laboratory for Optical and Computational Instrumentation, New York) image processing program.

### Atomic Force Microscopy

The nanofibril dispersions of TO-SWNF, TO-HWNF, and TO-CNF were diluted to 0.01 wt % consistencies and dropped on freshly cleaved mica plates to let dry at ambient conditions. The morphology of nanofibrils was observed under an atomic force microscope (AFM) Veeco Multimode Nanoscope V (Bruker, Santa Barbara, CA) operating in tapping mode using a standard silicon cantilever (TESPA-V2, Bruker) with a spring constant of 42 N/m. Scanned AFM height images were analyzed using Gwyddion software,^49^ after mean plane subtraction and horizontal scar correction. A total of hundred fibers were averaged to plot the nanofiber size distribution plots for all samples.

### Fourier-Transform Infrared Spectroscopy

FTIR analysis of the networks was performed using a Nicolet Summit Everest with diamond attenuated total reflectance (ATR, Thermo Fischer Scientific Inc., Waltham, MA). Networks were scanned 64 times between 400 and 4000 cm^–1^ wavenumbers at 4 cm^–1^ resolution.

### Light Transmittance Measurements

Light transmittance of the 0.1 wt % nanofibril suspensions and nanofibril networks (grammage 20) was measured using a Cary 5000 spectrophotometer equipped with a double monochromator incorporated with an R928 photomultiplier tube detector (UV–vis region) between 400 and 800 nm wavelengths.

### Mechanical Testing

The dry and wet networks were tested using a Shimadzu AG-X universal testing machine (Shimadzu Corp., Kyoto, Japan) in tensile mode at RT using a 1 kN load cell. A digital micrometer (Mitutoyo, Tokyo, Japan) was used to determine the thickness of the dry specimens, while the wet specimens were located in between two flat glass slips, and the thickness was measured gently using a digital caliper (Mitutoyo Scandinavia AB, Upplands Väsby, Sweden). Dry network specimens were prepared using a punch and were about 6 mm wide, 65 mm long, and 11–35 μm thick depending on the sample and were conditioned at 50% relative humidity (RH) for more than 48 h before testing. The span length and the strain rate of dry specimens were 20 mm and 2 mm min^–1^, respectively, and the pretest load was 0.5 N.

For the testing of wet networks, specimens were let in dH_2_O for 24 h at RT to reach equilibrium absorption and were 70 mm long and 400–800 μm thick, depending on the sample. The span length and the strain rate of wet specimens were 40 mm and 4 mm min^–1^, respectively, and the pretest load was 0.2 N.

At least three specimens were tested for both the dry and wet networks for reliability. The maximum strength at break was reported as the tensile strength. Elastic modulus was calculated from the slopes of linear elastic regions. The elongation was measured as the change in distance between grips divided by gauge length.

### Thermogravimetric Analysis (TGA)

The thermal stability measurements of the TO-SWNF, TO-HWNF, and TO-CNF networks were conducted using an STA 449 F3 Jupiter TGA (NETZSCH-Gerätebau GmbH Branch Office Scandinavia, Täby, Sweden). The analysis was performed at a heating rate of 10 °C min^–1^ from room temperature to 500 °C under an argon atmosphere.

### Water Absorption Capacity

Dry networks with grammage 20 g m^–2^ were immersed in dH_2_O at RT and allowed to swell for predetermined time intervals. Excess water on the surface of networks was dried by contacting sample surfaces softly on a partly wet tissue paper and then networks were weighed. The % water absorption capacity was calculated as percentage according to eq 3, where Wt is the total and Wd is the dry weight of the networks.
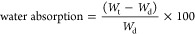
3

### Porosity Calculation

The porosity of the networks was calculated according to eq 4,^50^ using bulk density and water content values after conditioning samples in 50% RH at 23 °C for 48 h, where the true density for TEMPO-oxidized wood nanofibers was taken as 1.5 g cm^–3^, corresponding to the density of crystalline cellulose. A digital micrometer (Mitutoyo, Tokyo, Japan) was used to determine the thickness of the nanofiber networks for bulk density calculation.
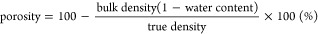
4

### Viscoelastic Properties

Compression–stress relaxation tests were performed on wet networks at equilibrium water absorption using a Discovery HR-2 rheometer (TA Instruments, New Castle, DE) with a protocol adjusted for materials with short relaxation times. An 8 mm parallel plate geometry was employed, and all measurements were carried out at 25 °C. Temperature was controlled by a Peltier element. Nanofibril network discs with ø 8 mm were prepared with the help of a biopsy punch, and the materials were incubated in Milli-Q water (18.2 MΩ cm^–1^) for at least 30 min prior to the test. The samples were sequentially compressed and allowed to relax. Compression to axial force levels of 0.1, 0.5, 1, 2, 4, and 6 N was performed with a compression speed of 5 μm s^–1^. Subsequently, the gap size was maintained constant while the samples were allowed to relax for 5 min. The viscoelastic properties were assessed in this step with small amplitude oscillatory deformations at a frequency of 1 Hz and 0.01% strain (*i.e.*, in the linear viscoelastic region). The samples were run in triplicate.

### Toxicological Risk Assessment

TO-SWNFs, TO-HWNFs, and TO-CNFs were evaluated on the toxicological risk assessment with human primary keratinocytes and fibroblasts. Cells were isolated from healthy patients undergoing routine reduction surgeries at Linköping University Hospital, Sweden. Procedures were performed under ethical approval from the Swedish Ethical Review Authority (2018/97/31). Samples were processed according to a modification of the protocol described by Rheinwald and Green.^51^ Briefly, subcutaneous fat was mechanically removed and the remaining tissue was enzymatically digested overnight. Dulbecco’s modified Eagle’s medium (DMEM; Gibco Thermo Fisher Scientific, Paisley, U.K.) containing 2.5 mg mL^–1^ dispase (Gibco Thermo Fisher Scientific) with a +4 °C overnight incubation was used for keratinocytes, and DMEM containing 165 U mL^–1^ collagenase (Gibco Thermo Fisher Scientific, Paisley, U.K.) and 2.5 mg mL^–1^ dispase with a 37 °C, 5% CO_2_, and 95% humidity incubation was used for fibroblasts. Thereafter, followed a 15 min incubation in DMEM containing 0.02% versine and 0 1% trypsin (Gibco Thermo Fisher Scientific, Paisley, U.K.). Isolated keratinocytes were seeded into 75 cm^2^ Corning culture flasks (Merck Life Sciences AB, Solna, Sweden) with Keratinocyte Serum-Free Medium (KSFM; Gibco Thermo Fisher Scientific, Paisley, U.K.) containing 1 mg L^–1^ epidermal growth factor, 25 mg L^–1^ bovine pituitary extract, 50 U mL^–1^ penicillin, and 50 mg mL^–1^ streptomycin (Gibco Thermo Fisher Scientific). Isolated fibroblasts were seeded into 75 cm^2^ culture flasks with DMEM containing 10% fetal calve serum (FCS; Gibco Thermo Fisher Scientific), 50 U mL^–1^ penicillin, and 50 mg mL^–1^ streptomycin. Medium was changed 3 times per week until confluency was reached.

Following the establishment of primary cultures, cells were enzymatically detached using 0.02% versene/0.1% trypsin and seeded in flat-bottomed 96-well plates (Falcon, Corning Inc.). Moreover, the samples were autoclaved prior to use at 121 °C for 20 min. Cells were allowed to adhere for 48 h before treatment with 0.01% TO-HWNF, TO-SWNF, and TO-CNF (*n* = 3–6). Cells were incubated for 48 h and continuously monitored using a LiveCyte 2 kinetic cytometer (Phase Focus Ltd. Sheffield, U.K.). Ptychographic images were captured every 20 min. Data was exported and analyzed using PF Assay Analysis v.3.7.1 (Phase Focus Ltd.). Cell counts and speeds for all treatment groups were exported to Prism 8.0 (Graphpad, LaJolla) for statistical analysis and generation of graphs. Cell counts in the treatment groups were normalized to nontreated controls and expressed as a proliferative index. Cell proliferation was compared using a two-way analysis of variance (ANOVA) coupled with Dunnett’s post-test. Average cell speeds for each well, expressed in μm s^–1^, were compared using ANOVA coupled with Dunnett’s post-test. All values are plotted as mean ± standard deviation. *P* values <0.05 were considered statistically significant.

## Results and Discussion

### TEMPO-Oxidation of Softwood and Hardwood

**Figure 1:**
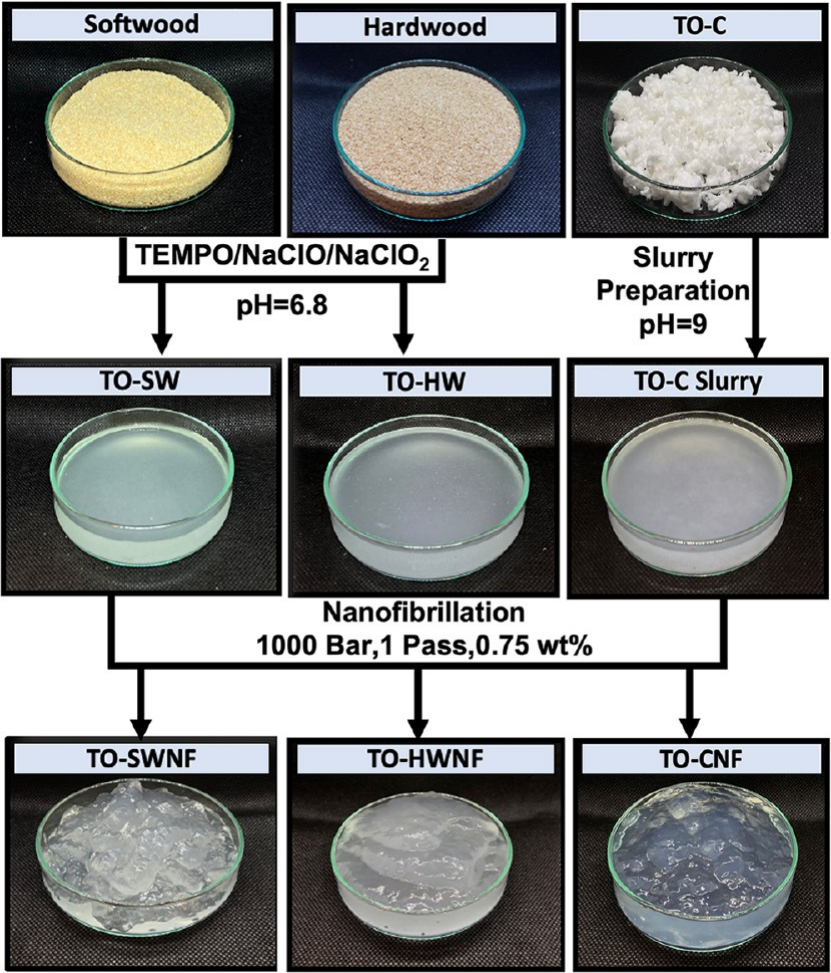
Photographs of the production steps of TO-SWNF, TO-HWNF, and TO-CNF.

Figure 1 shows the production process of TO-SWNF, TO-HWNF, and TO-CNF. Wood particles were directly TEMPO-oxidized without further size sectioning as shown in images, and TO-C was readily bought as the commercial TEMPO-oxidized pulp, visually recognizable by the white color compared to that of wood particles. After oxidation of SW and HW, the materials were visually comparable (Figure 1). After fibrillation all materials were observed as gels, with TO-CNF being visually the most transparent.

**Figure 2:**
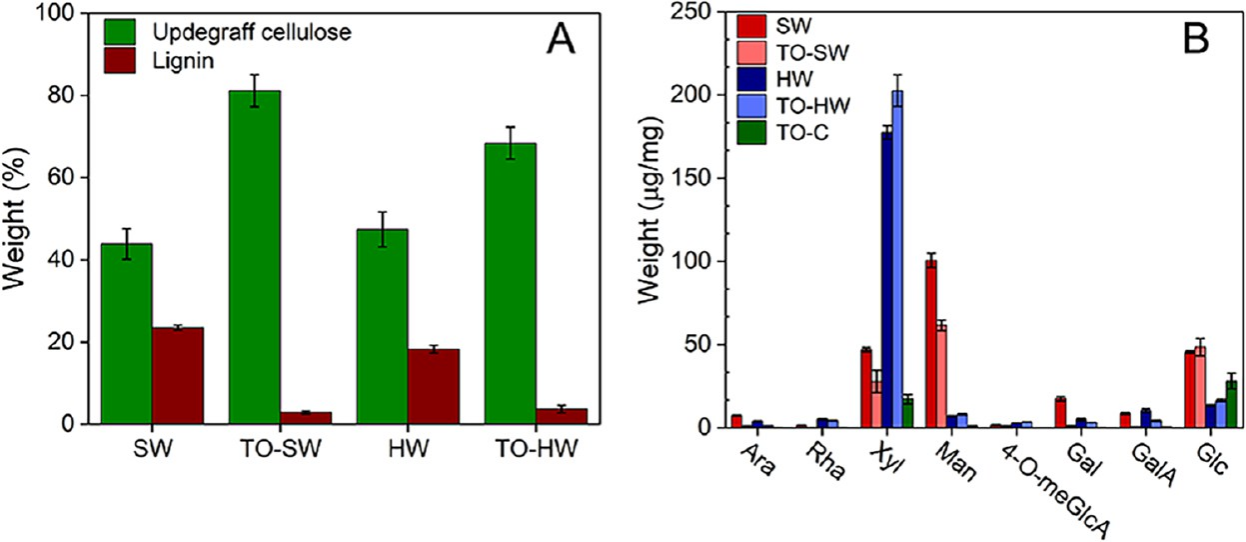
(A) Updegraff cellulose and lignin portions of SW, HW, TO-SW, and TO-HW. Lignin was determined using two different analytical methods, namely, Klason lignin assay for SW and TO-SW samples and acetyl bromide assay for HW and TO-HW samples. (B) Main hemicellulosic monosugar composition of the noncrystalline fractions of SW, HW, TO-C, and oxidized wood samples. Values belong to the noncrystalline polysaccharides in the cell wall of SW and HW, and the noncrystalline parts of TO-C, TO-SW, and TO-HW.

Updegraff cellulose and lignin portions of SW and HW before and after oxidation and monosugar composition of all samples are shown in Figure 2.

For TO-SW and TO-HW, the mass yields were 29 ± 2 and 48 ± 5 wt %, respectively. Compared to other hardwood species, the mass yield of TO-HW was higher than the reported yield of TEMPO-oxidized paulownia at pH = 10 (38 wt %),^24^ and similar to that of direct TEMPO-oxidized aspen (55 ± 1 wt %).^16^ In mild TEMPO-oxidation, depolymerization of cellulose chains at high alkaline conditions caused by β-elimination of glycosidic linkages are eliminated,^15^ which might have contributed to the higher mass yield of TO-HW.

From Figure 2A, an increase in the crystalline portion of both SW and HW after direct oxidation regarding delignification as well as the elimination of amorphous constituents of the cell wall was observed. Crystalline cellulose portions were found to be 47.4 ± 4.3 wt % for HW and 68.4 ± 3.9 wt % for TO-HW, whereas SW and TO-SW had 43.9 ± 3.7 wt % and 81.1 ± 3.9 wt %, respectively. The results demonstrate that TO-SW contained more crystalline cellulose and a smaller portion of amorphous hemicelluloses after oxidation in comparison to TO-HW. The pyrolysis/GC-MS data revealed lignin amounts lower than 3% for TO-C, and it was thus considered nearly lignin free. A major degradation in lignin during the oxidation of HW and SW was observed from 18.3 to 3.7 wt % for HW and TO-HW, and from 23.5 to 2.9 wt % for SW and TO-SW.

Figure 2B shows the main hemicellulosic monosugar proportions of SW, HW, TO-C, and oxidized wood samples, where the sugar contents are normalized against wood dry weight and TEMPO-oxidized wood dry weight, respectively. SW contained high amounts (μg/mg of sample) of galactose (17.4), glucose (45.5), and mannose (100.5), which reflects the most abundant hemicellulose galactoglucomannans in SW (Figure 2B and Table S1). Xylose (47.0) and arabinose (7.4), mainly derived from arabinoglucuronoxylans were the other prominent sugars in SW. In comparison, HW contained more xylose (177.4) than SW (47.0), while the opposite trend was observed for mannose. A decrease in the proportion of xylose and mannose was observed when SW was oxidized, whereas in HW, the proportion of xylose increased slightly after oxidation. The bond between uronic acids and xyloses of the dominating hemicellulose *O*-acetyl-(4-*O*-methylglucurono)xylan of HWs is known to be resistant to degradation through acid hydrolysis,^18^ and it may also explain the retained xylose in TO-HW. The increase in glucuronic acid monosugar (GlcA) was distinct for SW and TO-SW (Figure S2, Supporting Information), indicating a higher amount of GlcA units for noncrystalline polysaccharides in TO-SW contributing to the measured number of carboxylate content in titration. The same trend was not observed in the case of HW and TO-HW, indicating that the determined carboxylate content for TO-HW was likely on the surface of the crystalline cellulose portions (Figure S2, Supporting Information). For TO-C, glucose and xylose were the main detected sugars.

**Figure 3:**
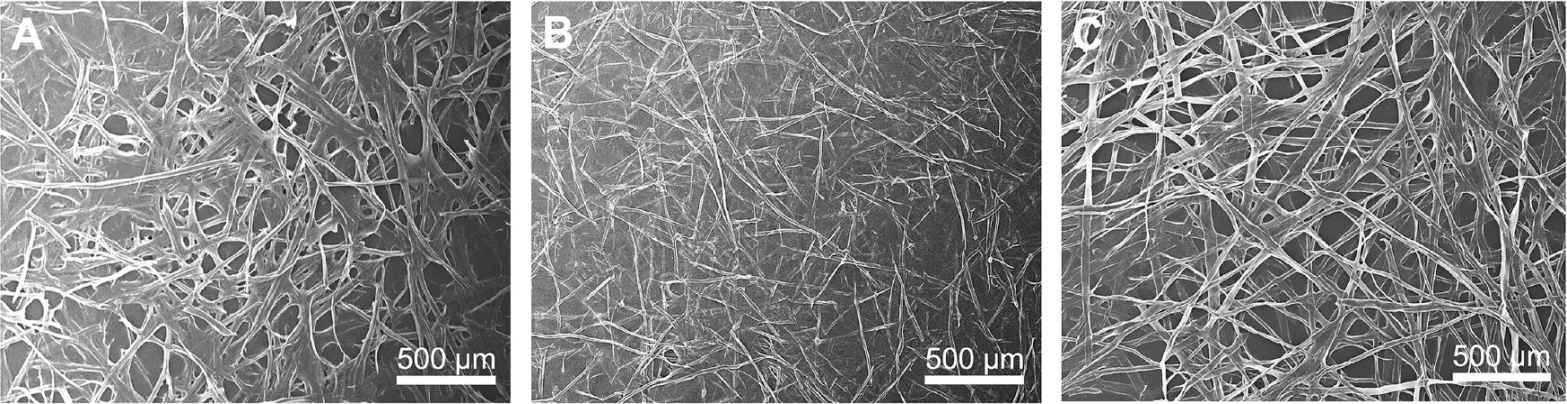
SEM images of (A) TO-SW, (B) TO-HW, and (C) TO-C fibers prior to fibrillation.

The SEM images of the TO-SW, TO-HW, and TO-C fibers prior to fibrillation are shown in Figure 3. TO-HW morphology as observed in Figure 3B shows rigid fibers with varying lengths including fibers smaller than 2 mm with detectable end points. TO-SW (Figure 3A) and TO-C (Figure 3C), which both are fibers of SW species, exhibited similar morphologies with fiber ends being difficult to discern and overall appearing to be longer compared to TO-HW.

### Nanofibrillation

**Figure 4:**
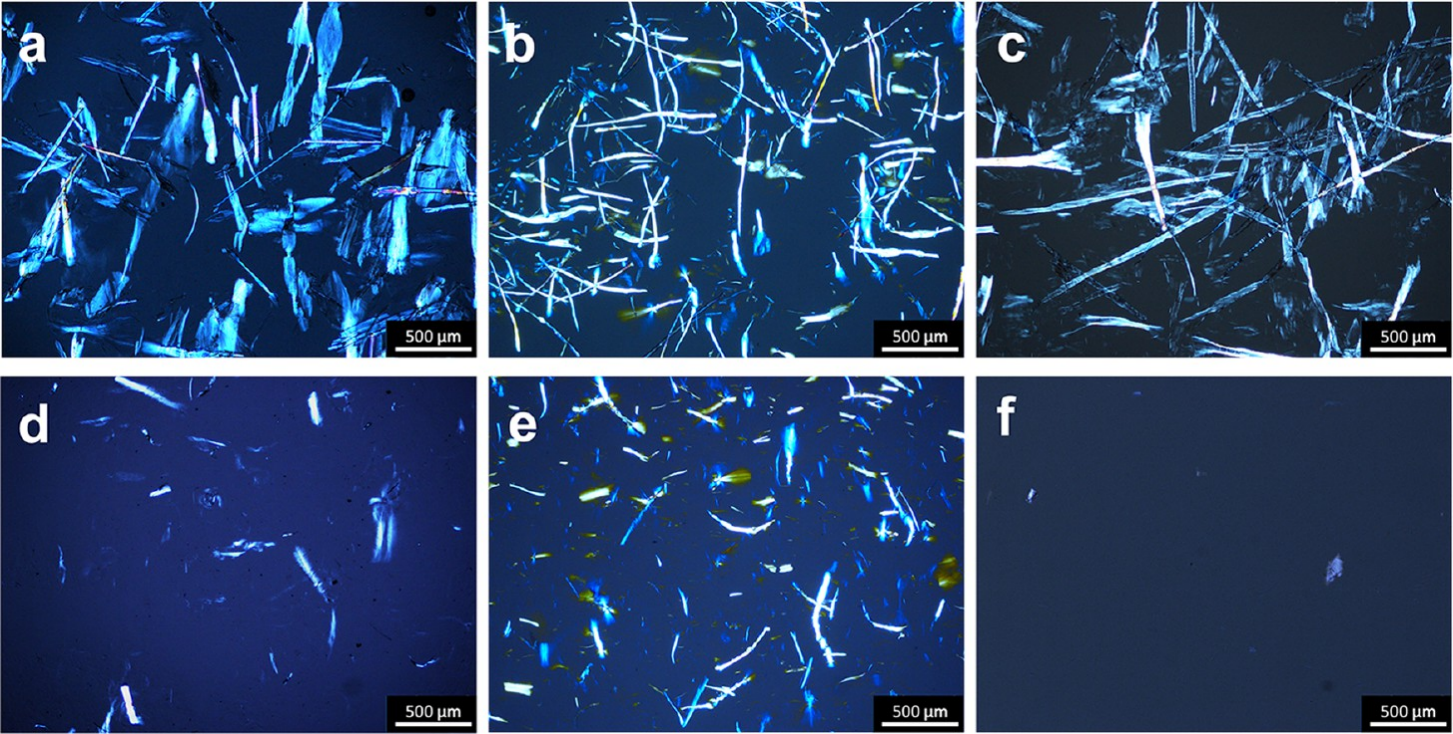
Polarized optical micrographs of (a) TO-SW, (b) TO-HW, (c) TO-C, and (d) TO-SWNF, (e) TO-HWNF, and (f) TO-CNF. Scale: 500 μm.

TO-SW, TO-HW, and TO-C were nanofibrillated using a microfluidizer. Figure 4 shows the POM images of fiber dispersions before and after fibrillation. Prior to fibrillation, all samples exhibited microfibers with lengths exceeding 500 μm. After fibrillation, TO-SWNF and TO-HWNF still contained microfibers detectable in the μm range. However, the degree of fibrillation appeared to be greater for TO-SW than for TO-HW, as evidenced by fewer large structures in POM images at the same concentration (Figure 4d,4e). Following fibrillation, almost no structure was detectable in POM for the TO-CNF sample (Figure 4f), suggesting a higher degree of fibrillation of TO-CNF compared to that of TO-SWNF and TO-HWNF.

The carboxylate contents of TO-SWNF, TO-HWNF, and TO-CNF were calculated from conductometric titration curves as 0.46 ± 0.05, 0.52 ± 0.04, and 1.20 ± 0.04 mmol g^–1^ of dry nanofibril, respectively. The lower carboxyl content of TO-SWNF and TO-HWNF in comparison to that of TO-CNF is associated with the consumption of oxidants NaClO/NaClO_2_ for degradation of lignin as well as the oxidation of cellulose in wood species. The slightly higher carboxylate content of TO-HWNF than TO-SWNF agrees with the direct TEMPO-oxidation applied within poplar genotypes with varying lignin contents, where lower content of lignin resulted in higher carboxylate content.^25^ Since the anatomy of SW is distinct from that of HW, the overall lignin structure will also affect lignin degradation kinetics through hydrolysis, which would differ for SW and HW species.^52^

Viscosities of TO-SW, TO-HW, and TO-C before and after fibrillation are shown in Table 1. For all samples, the viscosity was increased after fibrillation as a result of an enhanced fiber interaction.

**Table 1:** Viscosity Measurements before and after Fibrillation

materials	viscosity before (mPa·s)	viscosity after (mPa·s)
TO-SW	3.8 ± 0.4	2077 ± 55
TO-HW	3.7 ± 0.5	749 ± 63
TO-C	1.7 ± 0.1	242 ± 13

The viscosity of CNF suspensions is related to several factors such as surface chemistry, chemical composition of the material, and fiber aspect ratios,^53^ and showed a variance for TO-SWNF, TO-HWNF, and TO-CNF. TO-SWNF viscosity with 2077 ± 55 mPa·s was considerably higher than TO-HWNF with 749 ± 63 mPa·s, indicating a higher aspect ratio for TO-SWNF.^54^ In another study, it was proposed that hemicelluloses help in maintaining dispersion stability and increase viscosity of CNF in aqueous state,^55^ which might also have been the case for TO-SWNF and TO-HWNF with preserved hemicelluloses (Table S1). The lower viscosity of TO-CNF with higher carboxylate content was likely linked to the reduced length of the TO-C fibers, resulting in a decrease in aspect ratios and flocculation. In the case of TO-CNF, agglomeration was less likely due to a higher charge thus repulsion between the nanofibrils, and fiber contact tendency was lower due to lower aspect ratio in aqueous gel state.^56^

Both TO-SW and TO-HW fibers were introduced to the microfluidizer after washing at nearly neutral pH. Preserved hemicelluloses in TO-SW and TO-HW after oxidation (Table S1) might have facilitated fibrillation *via* hindering the H-bonding of microfibril aggregation, recognizing that cohesive mechanism of cellulose fibrils involves not only H-bonds but also ionic interactions, London dispersion, and electrostatic multipole interactions.^57^ The commercial TO-C was carboxylic acid type and consisted of a large number of glucuronic acid units with low p*K*_a_ values. Considering this, prior to fibrillation, NaOH was added until the pH of the slurry was adjusted to 9, with the assumption that sodium carboxylate groups on fiber surfaces were created. The concentration of all slurries was 0.75 wt % before fibrillation and was higher than the concentrations used in the literature for TO-C^50^ and direct oxidized aspen,^16^ which allowed processing of more materials in the microfluidizer at once. Figure S4 shows the images of the 48 h stability test on nanofiber suspensions with no visual sedimentation, indicating well-dispersed and stable fibers in the gel state after fibrillation.

**Figure 5:**
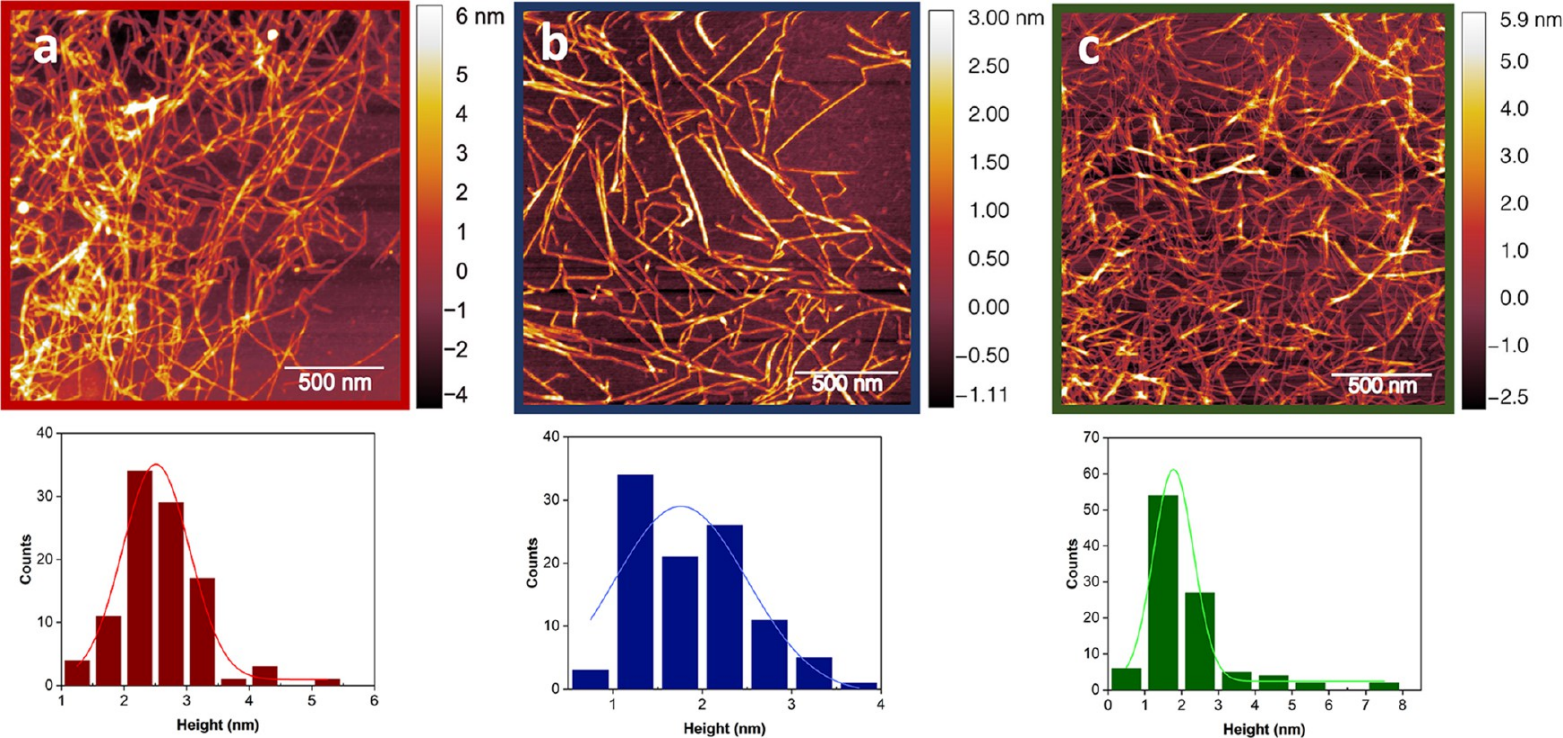
AFM height images and corresponding height distribution profiles of (a) TO-SWNF, (b) TO-HWNF, and (c) TO-CNF.

Figure 5 shows AFM images of nanofibrils with fiber heights smaller than 10 nm for all specimens. Average height values for TO-SWNF, TO-HWNF, and TO-CNF were 2.5 ± 0.7, 1.9 ± 0.7, and 2.5 ± 1.4 nm, respectively (Figure 5). The average height measured for TO-HWNF agreed with the nanofibril height (1.6 ± 0.6 nm) previously reported for directly oxidized aspen.^16^ Interestingly, the average height of the TO-HWNFs was found to be smaller than that of TO-SWNFs. Overall, these values were smaller than 3–4 nm for all samples, corresponding to the width of an elementary cellulose nanofibril. Elsewhere, the possibility of delamination of elementary cellulose fibrils during TEMPO-oxidation was studied by solid-state ^13^C nuclear magnetic resonance.^58^ In relation to that, the measured smaller average height was thought to be related to the oxidation of cellulose chains in elementary fibrils, resulting in a cleavage effect with widths around 2 nm. TO-C fibers were overall longer than 2 mm in pre-fibrillation SEM images (Figure 3C), where post-fibrillation nanofibrils shorter than 1 μm were present in TO-CNF (Figure S3). This suggests that fibrillation of TO-C might have yielded in vertical cutting of the fibril length for a portion of TO-C. On that note, the material used in the production of TO-SWNF and TO-HWNF networks also contains a portion of residual microfibers after fibrillation as observed from POM images (Figure 4). However, the AFM images shown in Figure 5 are only representative of the nanofibril fractions.

### Network Characteristics

Networks were prepared from TO-SWNF, TO-HWNF, and TO-CNF materials by using vacuum filtration and evaluated for characteristics essential in wound dressing applications. The functionality and conformability of wound dressings are important features connected to the material properties and thickness. The grammage of networks was chosen as 20 g m^–2^ adapted from previous optimizations considering the conformability of hydrogels onto skin with pliability.^38^ Material characteristics of the networks are summarized in Table 2. TO-SWNF and TO-HWNF networks exhibited higher porosity, where with increased degree of fibrillation of TO-C the film density was also increased. The network thickness decreased with increased fibrillation, and TO-CNF networks consisting of only nanofibrils exhibited the highest packing and smallest thickness values.^22^

**Table 2:** Material Characteristics of the Prepared Dry Networks Tested under Standard Conditions

materials	thickness (μm)	moisture content (%)	bulk density (g·cm^–3^)	porosity (%)
TO-SWNF	21.0 ± 1.0	8.8 ± 0.5	0.9 ± 0.0	48.5 ± 1.6
TO-HWNF	26.0 ± 2.0	9.8 ± 1.3	0.7 ± 0.1	58.1 ± 4.4
TO-CNF	15.0 ± 1.0	5.6 ± 3.3	1.4 ± 0.0	19.5 ± 5.6

**Figure 6:**
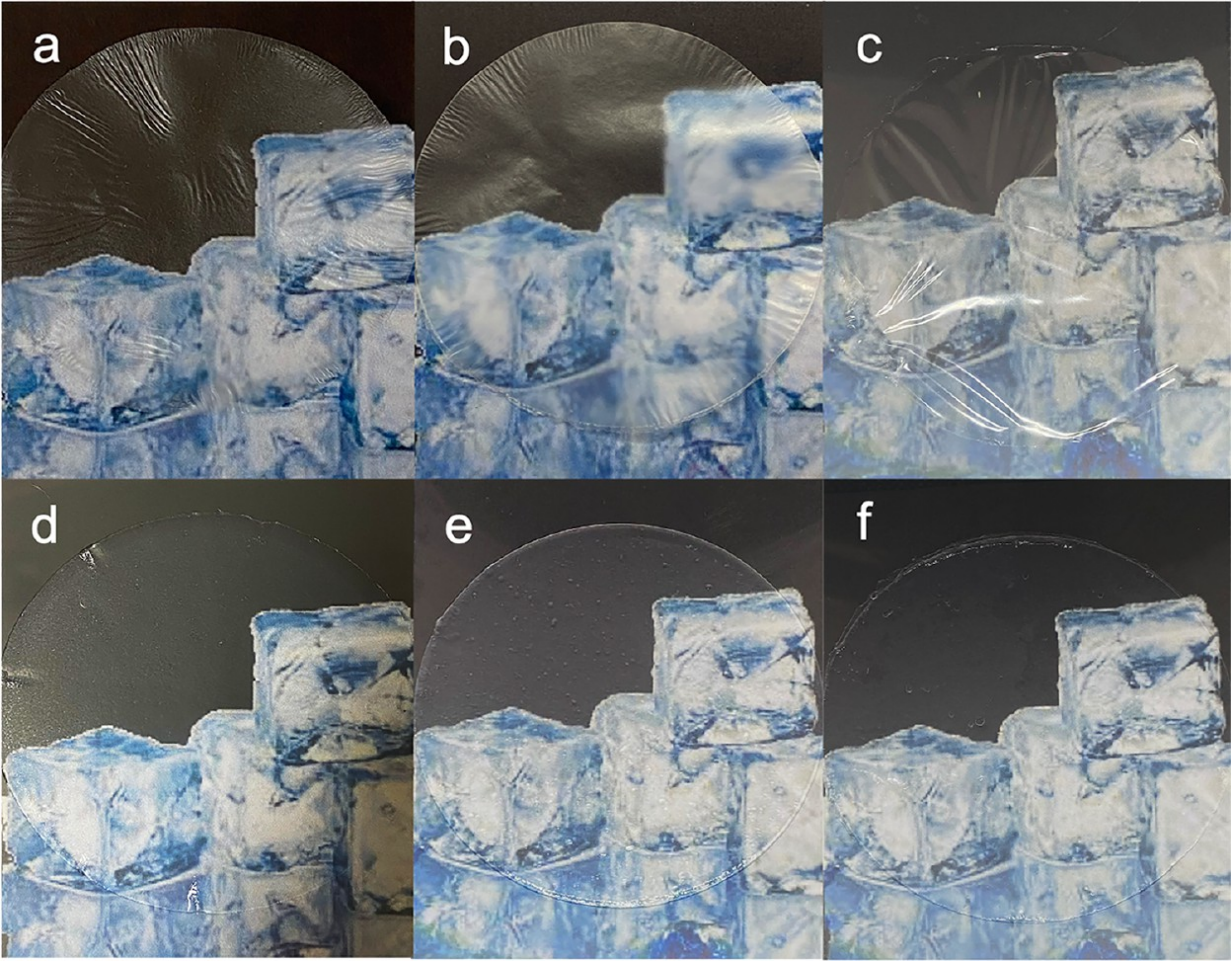
Photographs of (a) TO-SWNF, (b) TO-HWNF, (c) TO-CNF as dry networks, and (d) TO-SWNF, (e) TO-HWNF, and (f) TO-CNF as water-absorbed networks.

Another important feature is the transparency of dressings, which allows one to reveal the state of the wound and examine the healing progress without needless removal. Figure 6 shows the images of networks in dry (Figure 6a–c) and wet (Figure 6d–6f) states.

TO-SWNF and TO-CNF networks exhibited transparency as both dry and wet networks. In dry state, TO-HWNF networks revealed a slightly translucent appearance, which could be explained by the larger structures observed in the POM images (Figure 4e), and possible scattering of nonoxidized xylans^21^; nevertheless, they were transparent in both conditions. The transmittance of TO-SWNF networks was higher than that of TO-HWNF networks between 400 and 800 nm, also indicating a higher transparency (Figure S9).

**Figure 7:**
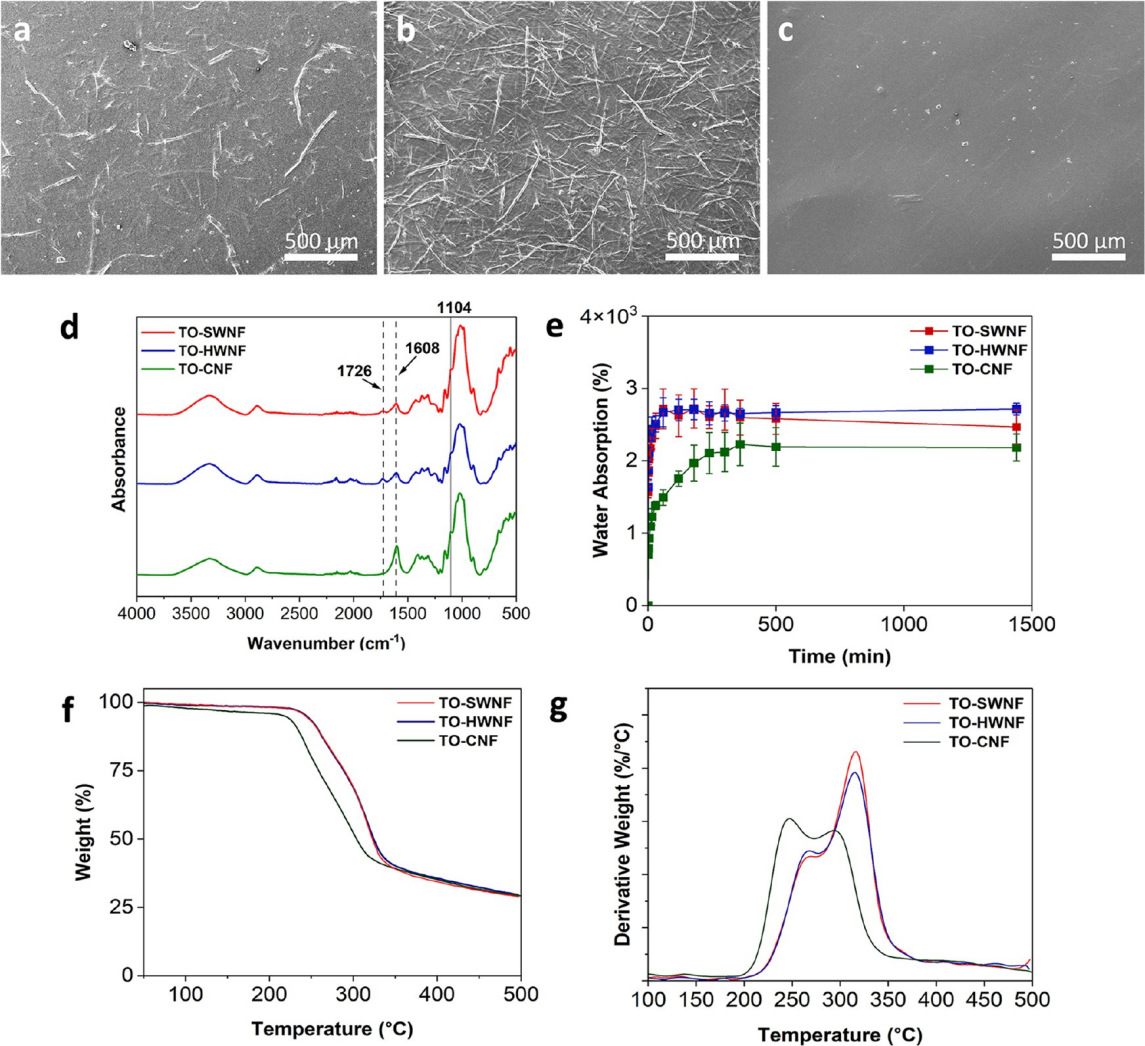
SEM surface images of (a) TO-SWNF, (b) TO-HWNF, and (c) TO-CNF networks. (d) FTIR spectra, (e) water absorption profiles, (f) mass loss curves, and (g) derivative weight curves of networks.

Figure 7 shows the surface SEM images, FTIR spectra, water absorption profiles, and TGA and derivative thermogravimetric (DTG) profiles of the networks. From the SEM images, many micro-sized fibers were detectable from the surface of TO-HWNF networks (Figure 7b). Microfibers were also detectable in TO-SWNF networks (Figure 7a), however to a much less extent compared to TO-HWNF. The surface of the TO-CNF (Figure 7c) network revealed almost no microstructure as expected, since it mainly consisted of nanofibrils with dimensions exceeding the visual range of SEM used in this study. FTIR of the dry networks confirmed the stretching vibrations of sodium carboxylate peak at 1608 cm^–1^ (reported 1603 cm^–1^),^23^ for both TO-SWNF and TO-HWNF networks (Figure 6d). The intensity of the peak at 1608 cm^–1^ was notably different for TO-CNF than for the TO-SWNF and TO-HWNF samples and represented the higher charge density of TO-CNFs. A peak at 1726 cm^–1^ belonging to the C=O stretching vibration from acetyl groups of hemicelluloses was detected for both TO-SWNF and TO-HWNF networks and was more evident for TO-HWNF, which was absent in the spectra of TO-CNF networks. The presence of hemicelluloses after oxidation could also be supported by TMS sugar analysis data presented in Table S1 and Figure S1. A peak at 1104 cm^–1^ associated with the C–O stretching of cellulose secondary alcohols can be observed in the spectrum of all networks.^59^

From a wound healing perspective, re-epithelialization is more rapid in a moist environment,^60^ with less scarring and reduced pain,^61^ and the absorption performance of dressing is correlated with removal of exudates from the site of wound. Hence, good absorption ability is crucial in the development of functional wound dressings. Figure 7e shows the water absorption profiles of the hydrogels, where the equilibrium absorbance was measured somewhere between 8 and 24 h for all materials. TO-SWNF and TO-HWNF exhibited very good water adsorption profiles, with values around 2466 ± 234 and 2713 ± 81% at 24 h equilibrium water absorption point, respectively. Interestingly, for TO-CNF networks that involved a higher number of hydrophilic carboxylate sites on the fiber surface, this value was lower than those of TO-SWNF and TO-HWNF with 2182 ± 186%. This trend is plausibly related to the higher packing degree of TO-CNF networks, where water penetration is limited to the space between the layers of the homogeneously fibrillated nanofibrils with finer widths. We have previously shown the separation of layers during swelling in oxidized CNF networks,^38^ and in the current study, the intrafibrillar interaction was impaired drastically due to loss of fibril interactions as the TO-CNF network swelled. This resulted in loss of structural integrity of TO-CNF networks upon swelling, likely in relation to the mentioned separation between the layers of nanofibrils. TO-SWNF and TO-HWNF networks were prepared directly from partially nanofibrillated fibers (Figure 7a,7b), which contributed to the higher porosity of networks (Table 2) and perhaps created cavities in networks allowing water diffusion without loss of structural integrity. Another aspect affecting the water absorption behavior is the presence of hemicelluloses, where the ratio between cellulose and hemicellulose, in combination with different hemicelluloses found in softwood and hardwood, might affect the degree of swelling.^38^

Sterilization is vital for dressings since they directly contact the site of the wound; they should be free of microorganisms. A common sterilization method is through steam or dry heat application typically between 120 and 250 °C temperature span to denature the structural proteins in microorganisms; therefore, the dressings should be able to tolerate heat treatments.^62^Figure 7f shows the TGA profiles of the networks. TO-SWNF and TO-HWNF networks exhibited almost identical weight loss profiles with higher thermal stability in comparison to TO-CNF networks with degradation onset temperatures of 268, 262, and 228 °C, respectively. Formerly, it was shown that the presence of carboxylates leads to a decrease in the thermal degradation of original cellulose *via* decarbonation of anhydroglucuronate units;^23^ thus, a higher thermal stability of TO-HWNF and TO-SWNF networks with lower carboxylate content was expected. DTG profiles of TO-HWNF and TO-SWNF showed two distinct peaks at 268 and 314 °C and could be distinguished from TO-CNF showing two peaks at 247 and 295 °C (Figure 7g). The DTG peak of TO-CNF at 247 °C was assigned to the thermal degradation (*T*_d_) point of anhydroglucuronate groups, which likely lowered the *T*_d_ of original cellulose in the presence of high carboxylate contents and was observed as a shift in the DTG peak from 313 °C (original cellulose) to 295 °C. A similar shift was not observed for TO-SWNF and TO-HWNF networks possibly because of the substantially lower amount of carboxylate groups. TO-SWNF and TO-HWNF also involved microfibers after fibrillation, which might have contributed to higher thermal dissipation before degradation in comparison to TO-CNF networks comprising fibers with higher specific surface area. Both TO-SWNF and TO-HWNF networks demonstrated the necessary thermal stability for targeted sterilized dressing applications.

### Mechanical Properties

Table 3 shows the mechanical properties of the nanofibril networks in the dry state. The ultimate tensile strength of TO-CNF networks was 206 ± 28 MPa, and it is higher than the strength of TO-SWNF and TO-HWNF networks with 166 ± 20 and 135 ± 14 MPa, respectively. The aspect ratio of CNFs is a key factor related to the strength in their networks; hence, the presence of fibers with larger widths affecting fiber interaction in TO-SWNF and TO-HWNF networks (Figure 7a,b) has likely caused a lower strength compared to TO-CNF dry networks. The viscosity of TO-SWNFs was higher than that of TO-HWNFs (Table S1), suggesting a higher aspect ratio for TO-SWNFs, corresponding to a higher strength of networks thereof. Furthermore, TO-SWNF networks have a larger number of nanofibrils, as evidenced from SEM images (Figure 7a). For TO-HWNF, the high content of xylans might have moderated the interaction of CNFs in their dry networks. Meanwhile, the elongation at break values of TO-HWNF, TO-SWNF, and TO-CNF networks are comparable (Table 3).

**Table 3:** Mechanical Properties of Dry Nanofibril Networks

sample	tensile strength (MPa)	elastic modulus (GPa)	elongation at break (%)
TO-SWNF	166 ± 20	7.5 ± 0.9	2.7 ± 0.3
TO-HWNF	135 ± 14	7.4 ± 0.6	3.6 ± 0.4
TO-CNF	206 ± 28	11.9 ± 1.9	2.5 ± 0.9

**Figure 8:**
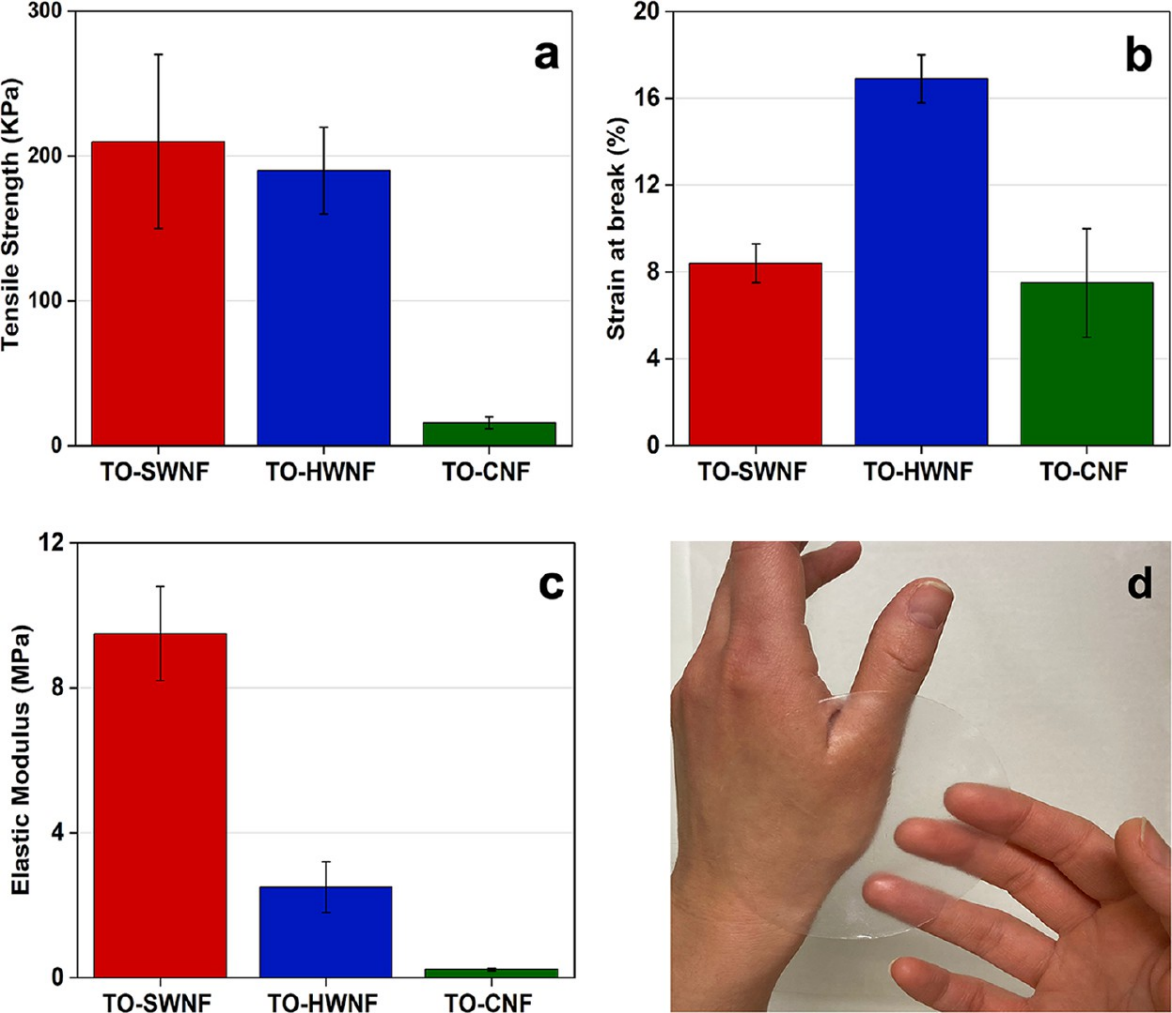
(a) Ultimate tensile strength, (b) elongation at break, and (c) elastic modulus of networks in wet conditions. (d) Photograph of wet TO-SWNF network applied on hand.

The assessment of mechanical performance in the wet state is of interest for potential CNF network wound dressings since the material is expected to maintain their structural integrity in wet state applications. Figure 8 shows the mechanical properties of wet networks under equilibrium water absorption conditions. All networks show a drastic decrease in strength and elastic modulus, while elongation at break increased in comparison to their dry state (Figures 8 and S5 and S6). The E-modulus of the TO-SWNF network was higher than that of the TO-HWNF and TO-CNF networks with 9.50 ± 1.30, 2.50 ± 0.70, and 0.25 ± 0.04 MPa, respectively. The significantly low modulus and strength of TO-CNF networks in the wet state show the effect of water on highly charged TEMPO network properties in wet applications. Water molecules are affecting the stress transfer ability of cellulose by disrupting closely interacted fibrils while competing with intrafibrillar H-bonds. This behavior was previously reported for TEMPO-oxidized cellulose nanofibril networks having ultimate tensile strength around 50 kPa in wet state.^63,64^ The high charge density on the TO-CNF surface is thought to increase the penetration of water molecules on the surface of individual fibrils, in comparison to TO-SWNF and TO-HWNF with fewer anionic sites on the fibril surface. In addition, we hypothesize that in the wet state, preserved hemicelluloses in TO-SWNF and TO-HWNF networks might have contributed to their higher strength and modulus by providing additional physical fibril–fibril interactions. Regarding the targeted application, mechanical performance of wet networks is important, and herein, oxidized SW and HW networks outperformed the TO-CNF networks in their maximum water absorption states.

**Figure 9:**
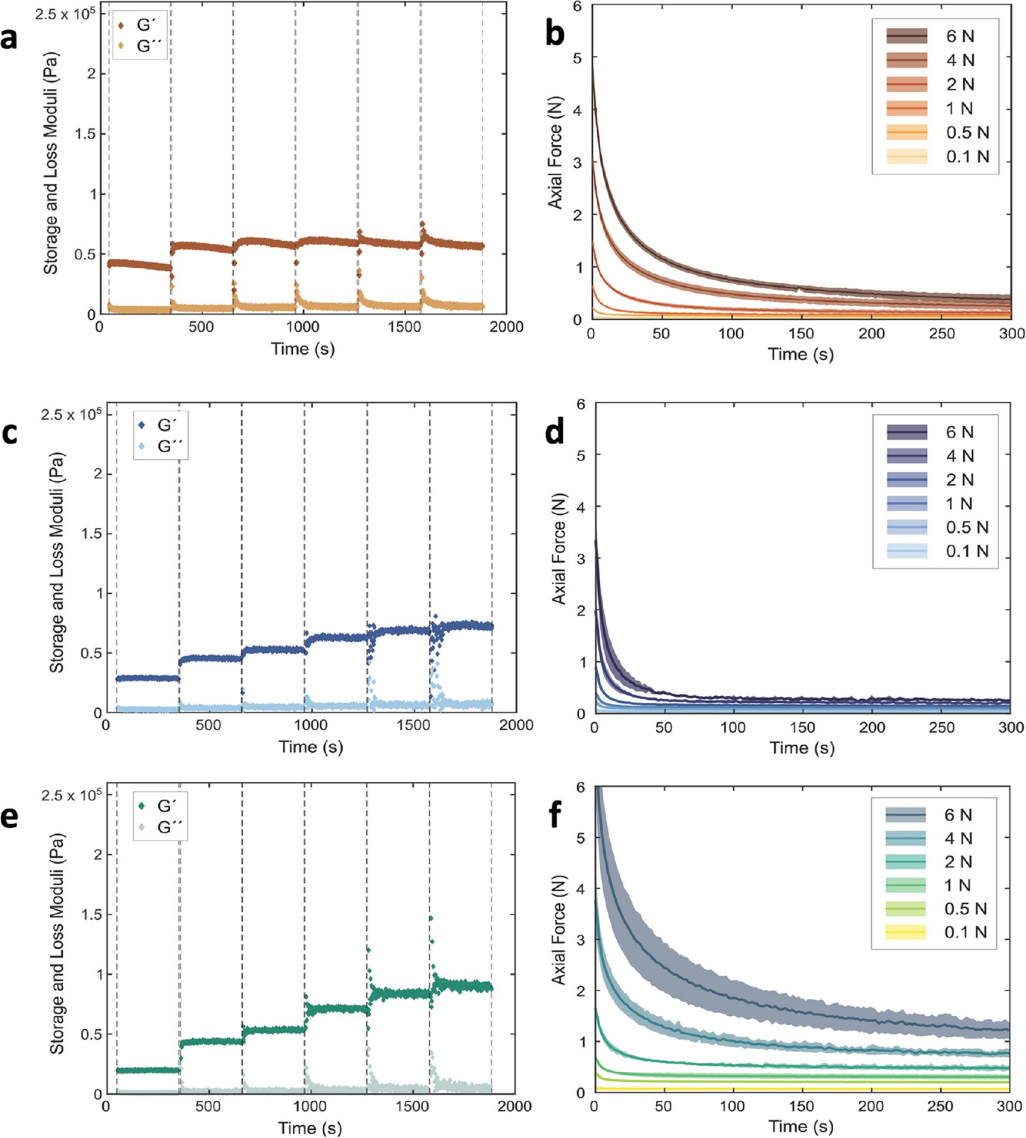
Storage and loss moduli of (a) TO-SWNF, (c) TO-HWNF, and (e) TO-CNF networks as a function of time. Axial force measurement of (b) TO-SWNF, (d) TO-HWNF, and (f) TO-CNF networks at equilibrium water absorption as a function of step time in the compression–stress relaxation test. Dashed lines in (a, c, e) correspond to the start and end of relaxation times for applied compression to axial force levels 0.1, 0.5, 1, 2, 4, and 6 N.

Figure 9 shows the viscoelastic properties of the wet networks assessed through compression–relaxation tests performed at equilibrium water absorption. The storage modulus (*G*′) increased from about 0.02 to 0.1 MPa with an increasing compression for all materials. The start and end values were comparable for TO-SWNF, TO-HWNF, and TO-CNF, and in the range of reported elastic modulus of *in vivo* skin tissue obtained through torsion and indentation tests;^65^ however, the dynamic response differed. TO-CNF exhibited the highest stiffening upon compression with more sites on fiber surfaces to create interfibrillar H-bonds, facilitated by the large number of carboxylate groups (Figure 9e). For TO-SWNF, an increase in *G*′ was seen for axial forces up to 0.5 N. Further compression did not result in any additional increase in *G*′, indicating that 0.5 N was sufficient to induce maximum interfibrillar interactions (Figure 9a). In contrast, *G*′ continued to increase upon axial compression for both TO-HWNF and TO-CNF but did eventually reach values similar to those of TO-SWNF (Figure 9c,9e). A correlation between the carboxylate contents and *G*′ of TO-SWNF and TO-HWNF networks was observed. The relaxation time was longest for TO-CNF and shortest for TO-HWNF at all applied forces (Figure 9b,d,f), likely owing to the larger number of carboxylate groups in TO-CNF resulting in more pronounced interfibrillar repulsion.

### Toxicological Risk Assessment

**Figure 10:**
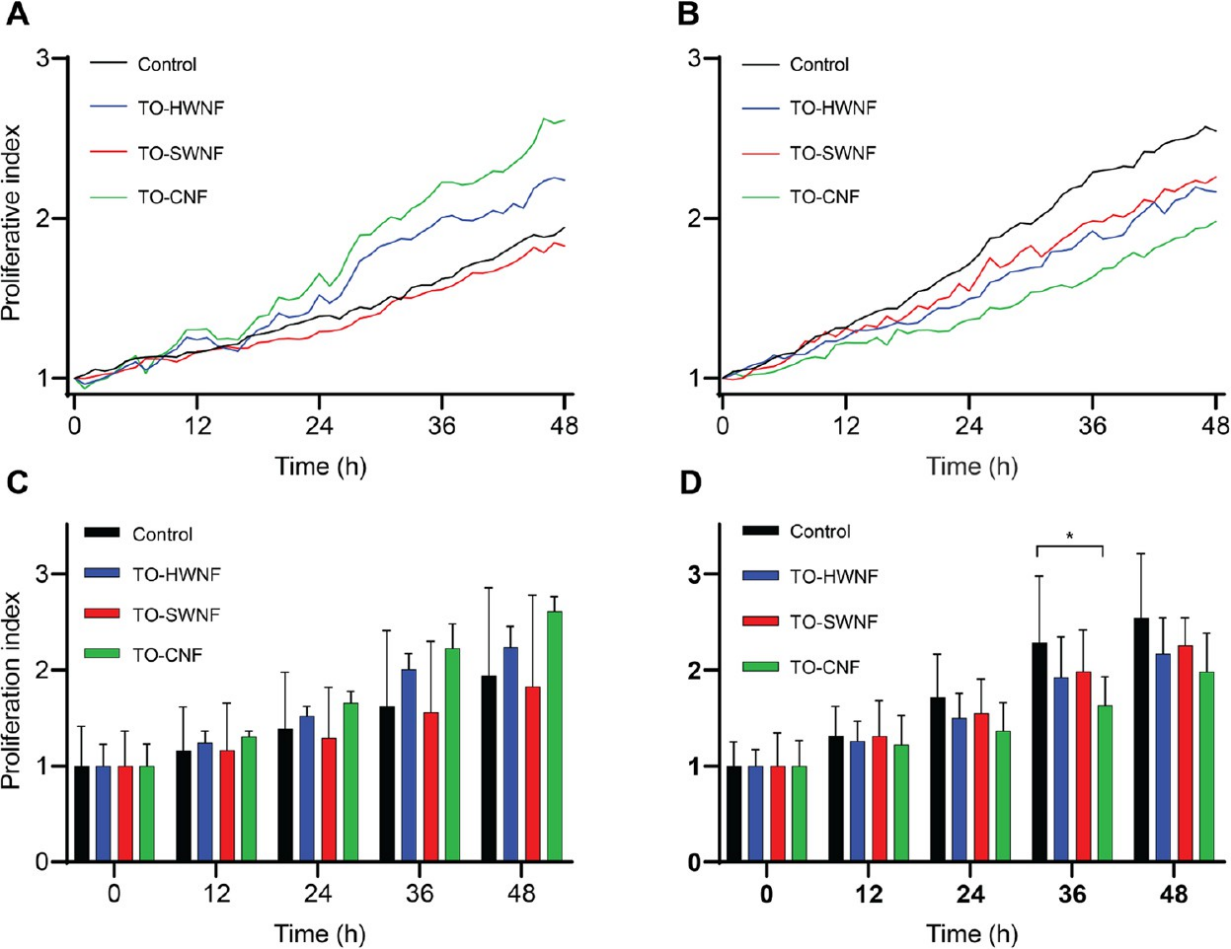
Graphs illustrating proliferation over time following exposure to TO-HWNF, TO-SWNF, and TO-CNF, as well as nontreated control. (A, C) Keratinocytes and (B, D) fibroblasts.

The possibility for the use of nanofiber networks toward wound dressings was evaluated through toxicological risk assessment on human primary keratinocytes and fibroblasts. Cells were exposed to TO-HWNFs, TO-SWNFs, and TO-CNFs for 48 h. No significant effects on cell proliferation compared to nontreated control was found, except for a significant reduction in fibroblasts treated with TO-CNF at 36 h (2.29 ± 0.69 for control and 1.63 ± 0.30 for TO-CNF, *p* < 0.05; Figure 10D). The assessments for the TO-SWNFs and TO-HWNFs are therefore considered to be without toxicological risk for applications intended for wound dressing.

**Figure 11:**
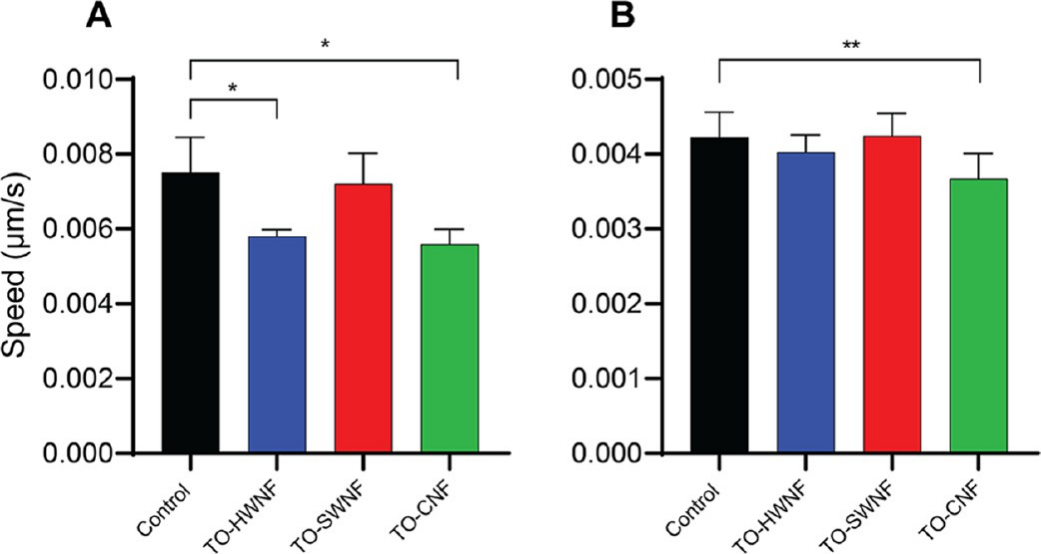
Graphs illustrating average cell speed over time following exposure to TO-HWNF, TO-SWNF, and TO-CNF, as well as nontreated control. (A) Keratinocytes and (B) fibroblasts.

Significant decreasing effects on average migratory speed were observed for keratinocytes treated with TO-HWNF and TO-CNF compared to nontreated controls (*p* < 0.05; Figure 11A) as well as fibroblasts treated with TO-CNF (*p* < 0.01, Figure 11B).

The cell response differs with CNFs with different surface characteristics in relation to structure, size, and surface charge.^28^ Particularly with fibroblasts, coatings of lower charge density TO-CNF were shown to improve fibroblast adhesion, spreading, and viability in comparison to coatings of TO-CNF with higher charge densities.^66^ A similar behavior is thought to exist in the case of TO-SWNF with the lowest charge density exhibiting the most control-like proliferative profile when tested with fibroblasts. Cell interaction and behavior are also related to mechanical stimuli, and there exist studies reporting fibroblasts having higher proliferation and spreading in stiffer gel matrices,^67^ as well as the contrary.^68^ In addition, the positive ions in the cell culture medium DMEM might have interactions with negatively charged nanofibrils and the hemicelluloses present in TO-SWNFs and TO-HWNFs; nevertheless, it is apparent that cell response cannot be linked to a single parameter. Since in this study the fibroblasts and keratinocytes demonstrated proliferation with all samples, we inferred that the mechanical properties of all nanofibrils were sufficient to support cell proliferation without toxicological effects. However, for further understanding, nanofibrils should be investigated in relation to their modulus of elasticity with DMEM and their cell proliferation behavior.

## Conclusions

In this work, cellulose nanofibrils were obtained by fibrillation of direct mild TEMPO-oxidation of commercial softwood and hardwood particles and were assembled into networks *via* vacuum filtration. Oxidized softwood and hardwood with 0.46 ± 0.05 and 0.52 ± 0.04 mmol g^–1^ respective carboxylate group contents were nanofibrillated by using a microfluidizer. Oxidized softwood nanofibril (TO-SWNF) and hardwood nanofibril (TO-HWNF) networks exhibited comparable mechanical properties to those of commercially bought and fibrillated TEMPO-oxidized pulp nanofibrils (TO-CNF) in dry state. All networks were transparent in wet state, while TO-SWNF (≈2500%) and TO-HWNF (≈2700%) exhibited high water absorption at equilibrium conditions. Directly oxidized wood networks showed superior mechanical properties to TO-CNF networks at equilibrium water absorption (16 ± 4 kPa), whereas TO-SWNF hydrogels (210 ± 60 kPa) exhibited the higher wet strength compared to TO-HWNF (190 ± 30 kPa). Toxicological risk assessment of TO-HWNFs and TO-SWNFs did not reveal significant negative effects regarding cell function, with TO-SWNFs having the lowest impact. However, TO-CNF did impair proliferation and migration to a small extent. Collectively, the results obtained in the present study demonstrate that cellulose nanofibril networks generated from commercially available softwood and hardwood particles using a straightforward production route have potential applications as wound dressings. In future work, the most promising hydrogels (TO-SWNF) will be tested *in vivo* toward possible real-life applications as wound dressings. Antimicrobial, barrier, and elastoplastic properties and the long-term stability of these networks are related to the potential application which also further needs to be investigated. The tunability of direct mild TEMPO-oxidation of different wood species in relation to varying oxidation parameters, such as time and amount of oxidizing agents, and their consequent CNF characteristics are interesting topics for future research as well.

## Supporting Information Available

Monosugar contents of SW, HW, TO-SW, TO-HW, and TO-C samples obtained from TMS monosaccharide analysis; glucuronic acid components of SW, HW, TO-SW, TO-HW, and TO-C samples; AFM image of TO-CNF (0.0015 wt %); photographs of suspension stability of TO-SWNF, TO-HWNF, and TO-CNF gels over 48 h; representative stress–strain curves of TO-SWNF, TO-HWNF, and TO-CNF networks in dry state; representative stress–strain curves of the TO-SWNF, TO-HWNF, and TO-CNF networks at equilibrium water absorption; drying process photographs of networks; transmittance of 0.1 wt % nanofibril suspensions; transmittance of 20 g m^–2^ nanofibril networks (PDF)
