# Supplementary material for: Preparation and Characterization of Softwood and Hardwood Nanofibril Hydrogels: Toward Wound Dressing Applications
Source: Biomacromolecules. 2023 Nov 11;24(12):5605–19. doi: 10.1021/acs.biomac.3c00596 (PMC10716857; doi:10.1021/acs.biomac.3c00596)
Supplement: Supplementary file 1 — bm3c00596_si_001.pdf [file bm3c00596_si_001.pdf]

Supporting Information for

Preparation and Characterization of Softwood and Hardwood Nanofibril Hydrogels – Towards Wound Dressing Applications

Yağmur Baş,[†] Linn Berglund,[†,] Totte Niittylä,[¤] Elisa Zattarin,[‡] Daniel Aili,[‡]*
Zeljana Sotra,[§] Ivana Rinklake,[§] Johan Junker,[§] Jonathan Rakar,[§] Kristiina Oksman[†,¥]

[†]Division of Materials Science, Luleå University of Technology, SE-971 87 Luleå, Sweden

[¤]Umeå Plant Science Centre, Department of Forest Genetics and Plant Physiology, Swedish University of Agricultural Sciences, Umeå, Sweden

[‡]Laboratory of Molecular Materials, Division of Biophysics and Biotechnology, Department of Physics, Chemistry and Biology, Linköping University, SE-581 83 Linköping, Sweden

[§]Center for Disaster Medicine and Traumatology, Department of Biomedical and Clinical Sciences, Linköping University, SE-581 85 Linköping, Sweden

[¥]Department of Mechanical & Industrial Engineering (MIE), University of Toronto, Toronto, ON M5S 3G8, Canada

*Linn Berglund − Division of Materials Science, Department of Engineering Sciences and Mathematics, Luleå University of Technology, SE-971 87 Luleå, Sweden; orcid.org/0000- 0002-6247-5963; E-mail: linn.berglund@ltu.se Phone: +46 (0) 920 493381

Table S1. Main hemicellulosic monosugar composition of the non-crystalline fractions of SW, HW, TO-C and oxidized wood samples.

**sample**	**xylose* (μg/mg)**	**glucose* (μg/mg)**	**arabinose* (μg/mg)**	**mannose* (μg/mg)**
SW	47.0 (1.2)	45.5 (0.7)	7.5 (0.2)	100.6 (4.3)
TO-SW	27.8 (6.6)	48.5 (5.2)	0.9 (0.2)	61.5 (3.1)
HW	177.4 (4.1)	13.3 (0.4)	3.9 (0.3)	6.9 (0.4)
TO-HW	202.5 (9.6)	16.4 (0.8)	1.3 (0.1)	8.1 (0.5)
TO-C	17.2 (2.9)	28.1 (4.8)	0.1 (0.0)	1.0 (0.2)

*Belongs to the non-crystalline polysaccharides in the cell wall of SW and HW samples.

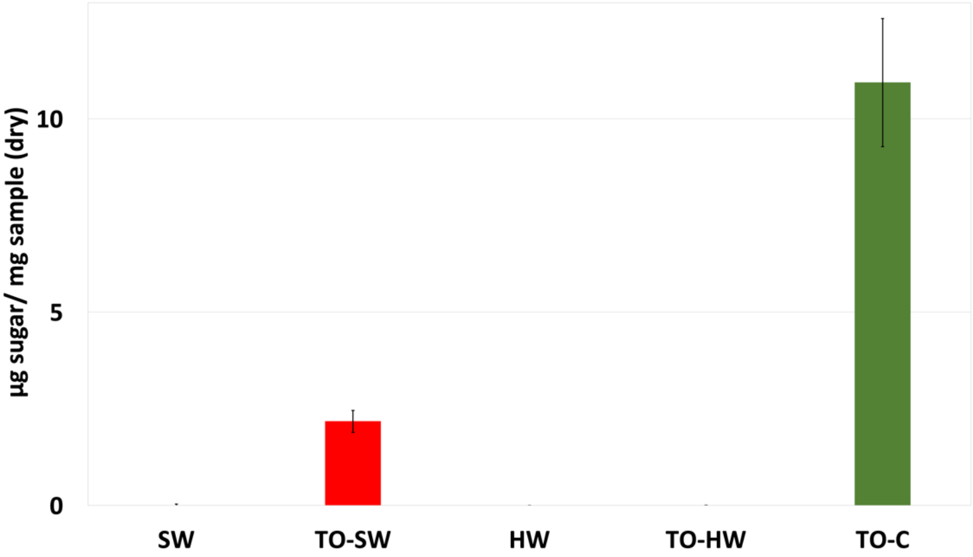

Figure S2. Glucuronic acid components of SW, HW, TO-SW, TO-HW and TO-C. Notice the increase of GlcA amount from SW to TO-SW is not observed in case of oxidation of HW, whereas GlcA component of TO-C was notably higher than TO-SW and TO-HW.

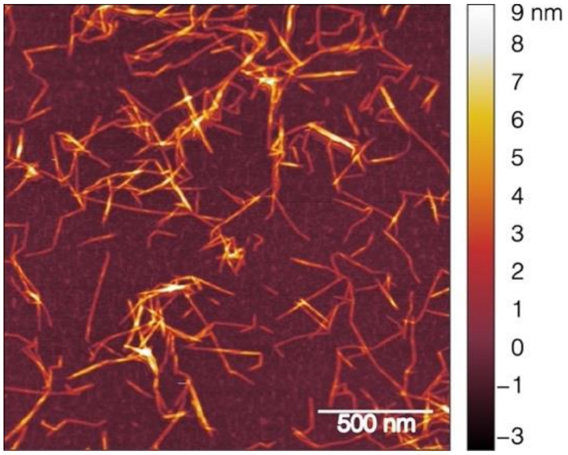

Figure S3. AFM image of TO-CNF. (Concentration 0.0015 wt %)

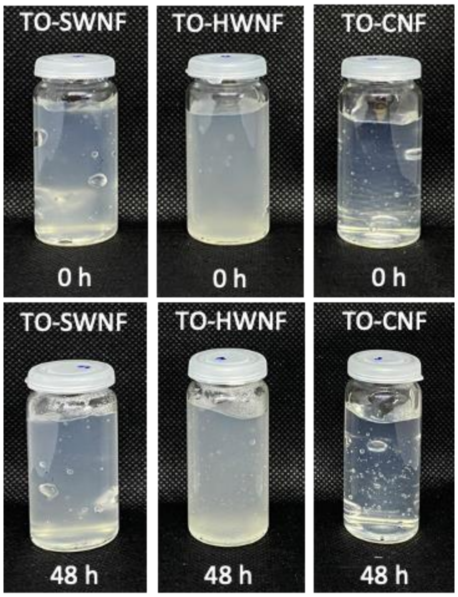

Figure S4. Suspension stability of specimens through 48 h. (Concentration: 0.75 wt%) After 48 h, no sedimentation was observed in the gels.

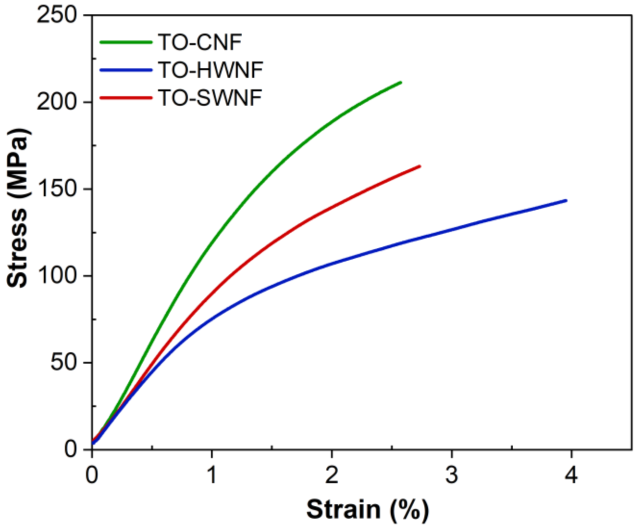

Figure S5. Representative stress-strain curves of dry networks. (20 g m^{-2})

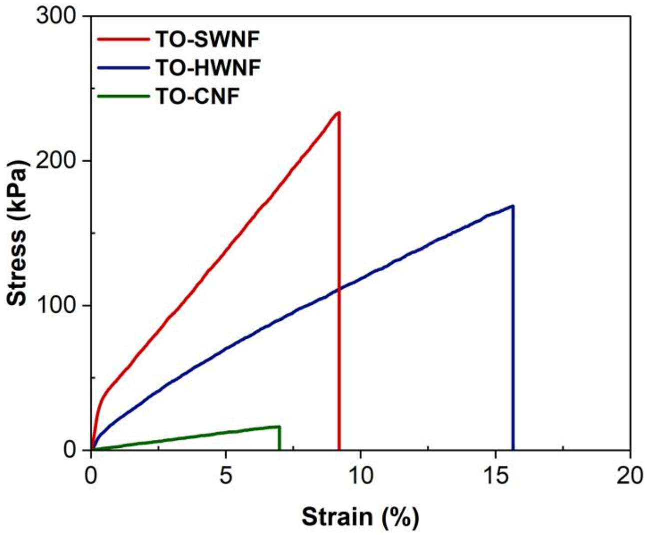

Figure S6. Representative stress-strain curves of the wet specimens at 24 h water absorption. (20 g m^{-2})

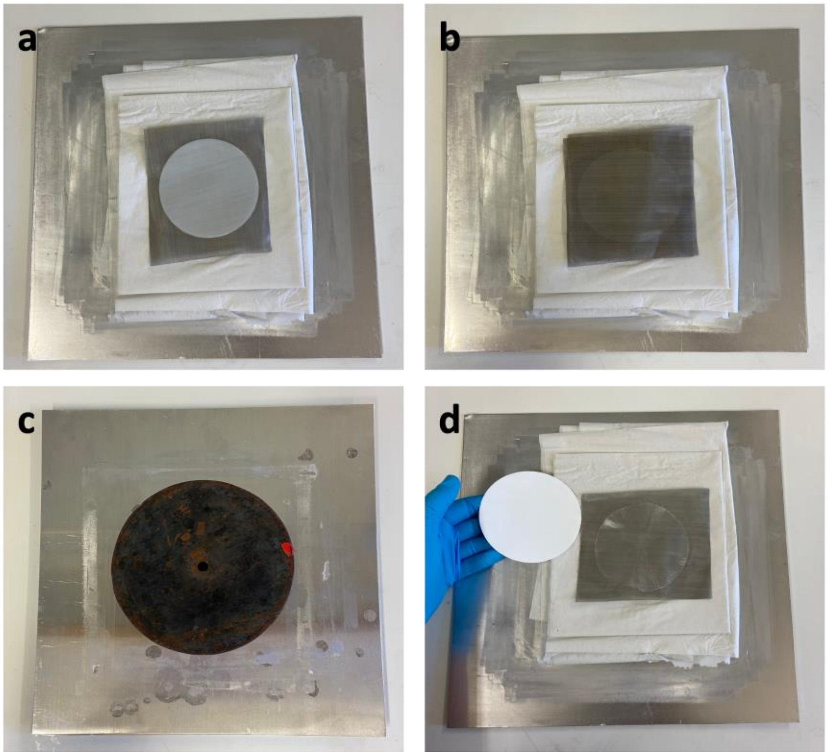

Figure S7. Images of drying process of the wet TO-SWNF cakes. a-b) Wet cake on membrane surrounded by metal meshes located in between paper tissues. c) Drying under a weight. d) Dried TO-SWNF network effortlessly separated from membrane surface.

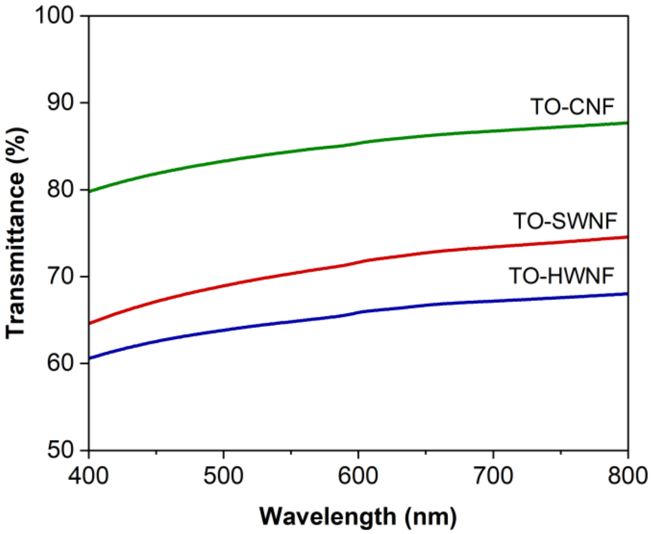

Figure S8. Transmittance of 0.1 wt% suspensions of nanofibrils.

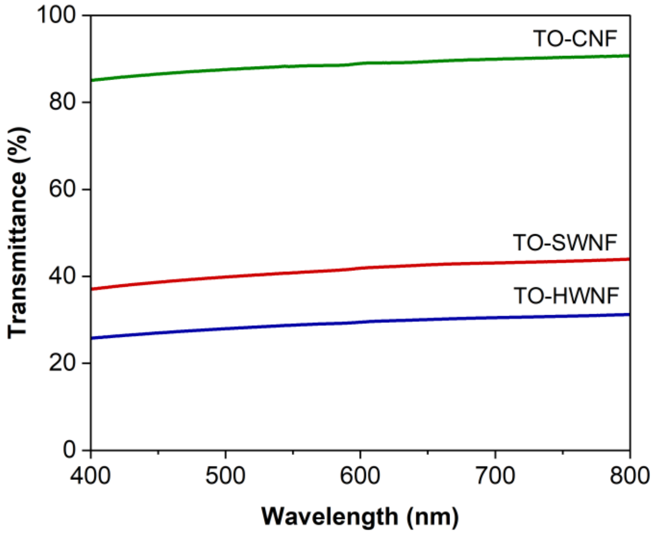

Figure S9. Transmittance of 20 g m^{-2} networks.
